# Comparative study of locking neutralization plate construct versus tension band wiring with a cannulated screw for patella fractures: experimental and finite element analysis

**DOI:** 10.1186/s13018-024-04538-w

**Published:** 2024-01-18

**Authors:** Sunjung Kim, Nirav Mungalpara, Rohan Wangikar, Majd Tarabichi, Joseph Karam, Asheesh Bedi, Jason Koh, Farid Amirouche

**Affiliations:** 1 Department of Orthopedic Surgery, University of Illinois at Chicago, Chicago, IL USA; 2 Department of Orthopedic Surgery, Orthopedic and Spine Institute, Northshore University Health System, An Affiliate of the University of Chicago Pritzker School of Medicine, 9669 Kenton Avenue, Skokie, IL 60076 USA

## Abstract

Transverse patella fractures, accounting for approximately 1% of Orthopedic injuries, pose intricate challenges due to their vital role in knee mechanics. This study aimed to compare the biomechanical performance of a construct, integrating cannulated screws and an anterior locking neutralization plate, with the conventional tension band wiring technique for treating these fractures. Experimental testing and Finite Element Analysis were employed to evaluate the constructs and gain profound insights into their mechanical behavior. Sixteen cadaveric knees were prepared, and transverse patella fractures were induced at the midpoints using a saw. The plate construct and tension band wire fixation were randomly assigned to the specimens. A cyclic test evaluated the implants' durability and stability, simulating knee movement during extension and flexion. Tensile testing assessed the implants' maximum failure force after cyclic testing, while Finite Element Analysis provided detailed insights into stress distribution and deformation patterns. Statistical analysis was exclusively performed for the experimental data. Results showed the plate enhanced stability with significantly lower deformation (0.09 ± 0.12 mm) compared to wire fixation (0.77 ± 0.54 mm) after 500 cycles (p = 0.004). In tensile testing, the construct also demonstrated higher failure resistance (1359 ± 21.53 N) than wire fixation (780.1 ± 22.62N) (p = 0.007). Finite Element Analysis highlighted distinct stress patterns, validating the construct's superiority. This research presents a promising treatment approach for transverse patella fractures with potential clinical impact and future research prospects. This study presents a promising advancement in addressing the intricate challenges of transverse patella fractures, with implications for refining clinical practice. The construct's improved stability and resistance to failure offer potential benefits in postoperative management and patient outcomes.

## Introduction

Patellar fractures, constituting approximately 1% of Orthopedic injuries, present unique challenges due to their intricate role in knee mechanics and joint function [1, 2]. While direct trauma and indirect forces can result in various patella fracture types, transverse fractures are the most prevalent [3, 4]. Initially considered a vestige, the patella is a mechanical pulley that enhances extensor forces by up to 30% [5]. This function is especially vital in the final 30 degrees of knee extension. Thus, the patella’s integrity is paramount for normal knee kinematics, underscoring the significance of optimal treatment.

Effective management of transverse patella fractures aligns with AO (Arbeit gemeinschaft für Osteosyntheses fragment) principles and aims to minimize hardware-related complications. Neglecting these fractures can result in a misalignment of the patellofemoral joint, potentially leading to early-onset patellofemoral osteoarthritis, restricted motion, and ongoing knee discomfort [6, 7].

Traditional methods such as using K-wires, screws, and cerclage wiring have been commonly employed among the treatment options for displaced patella fractures. While these conventional techniques have demonstrated effectiveness in many cases, they exhibit limitations when confronted with the distinctive biomechanical challenges observed in specific patient populations. Notably, older patients with osteoporosis or significant muscle atrophy present a unique set of circumstances that can compromise the outcomes of these standard approaches.

Treatment of transverse patella fractures in older patients or those with less muscle mass introduces additional complexities [8, 9]. The advanced age and potential for decreased bone density may lead to challenges in achieving stable fracture fixation and optimal healing. Complications such as implant failure, delayed union, or nonunion become concerns in these cases. Moreover, the increased load-bearing demands on the patellofemoral joint in elderly or osteoporotic patients with less muscle mass can place additional stress on the fixation hardware.

In specific patient populations, particularly those among the elderly with less muscle mass, the effectiveness of conventional methods comes under increased scrutiny. Notably, these cases often present factors such as variations in implant quality and anatomical considerations that can potentially impact treatment outcomes [10]. In response to these challenges, there is a growing interest in reinforcing fracture implants by adopting advanced techniques. One method that has gained attention is the anterior variable angle locking neutralization plate (VA LNP or construct). This approach is considered promising solutions to enhance implant stability, particularly in the complex scenarios posed by such patient groups. However, it is essential to highlight that a comprehensive biomechanical analysis of implants fortified with traditional TBW or an anterior VA LNP, mainly when used in conjunction with vertically oriented cannulated screws for transverse fracture fixation, is currently lacking in the existing body of literature.

The VA LNP, emerging as a promising treatment option, offers superior biomechanical support for patella fractures compared to tension band wiring. Bilateral VA LNP plating has demonstrated reduced interfragmentary displacement and higher failure loads compared to other methods under cyclic loading conditions [11, 12]. Moreover, VA LNP provides increased adjustability and stability of screw heads, offering improved outcomes compared to TBW [13, 14].

However, despite these advancements, the optimal treatment approach for transverse patella fractures remains a subject of ongoing investigation. This study explores the biomechanical advantage of an implant combining cannulated screws and VA LNP compared to TBW. We hypothesize that the hybrid VA LNP will exhibit higher load-to-failure properties and better fracture reduction maintenance under cyclic loading, presenting a promising alternative for treating transverse patella fractures.

Finite Element Analysis (FEA) has emerged as a valuable tool in investigating patellofemoral pressure dynamics. These studies aim to understand the intricate interplay between anatomical factors, loading conditions, and surgical interventions concerning patellofemoral pressure levels. While some research has focused on contact pressure distribution and cartilage stresses, validating FEA models against empirical measurements remains crucial [15, 16]. Few studies have explored parametric variations associated with symptomatic knees and treatment methods [17, 18]. Within the scope of our investigation, our study introduces a specialized FEA modeling technique. This technique is meticulously designed to evaluate the stress and deformation dynamics, particularly within the context of implant performance, explicitly emphasizing the maximum failure loading of the patellar tendon.

This study assesses the biomechanical superiority of a composite approach involving VA LNP compared to TBW. By comprehensively evaluating these two implant options' durability and tensile strength, we aim to validate our findings using FEA. Previous biomechanical inquiries into transverse patella fractures treated with a plate and tension band wire (TBW) have primarily involved experimental studies utilizing polyurethane forms. These studies predominantly focused on evaluating the behavior of the two principal fracture gaps while lacking the capacity for real-time sensing and monitoring [19]. Our research integrates FEA, primarily emphasizing early gap formation at the fracture level followed by load-to-failure testing and a comparative analysis with results obtained from cadaver experiments. Notably, cyclic testing was exclusively conducted within the cadaveric experiment and was not included in the FEA simulation.

## Method and materials

### Experimental testing

#### Specimen preparation

Sixteen knees were utilized, with a balanced distribution of left and right knees. The cadaveric specimens were obtained from human donors, comprising a diverse group of 10 female (63%) and 6 males (37%) [20]. All specimens were procured by donating organization (Science Care, Phoenix, Arizona, USA). The donor had no history of severe arthritis or missing ligaments and were evaluated by an orthopedic surgeon before their inclusion. This evaluation involved a multi-faceted approach: a thorough visual inspection was conducted to identify any signs of degenerative changes or osteochondral defects that are often not evident in medical history alone. Additionally, the surgeon performed manual testing to assess the integrity of ligaments, ensuring that they were intact and exhibited no significant wear or pathology.

The average age of the specimens was 78 years, ranging from 46 to 98 years. The mean body weight of the individuals was 151 pounds, with a weight range of 109 to 193 pounds. This demographic data is particularly relevant to our hypothesis, which states that patella fracture in older age requires more stability due to impaired bone quality and healing, age-related changes in immune signals and stem cell activity [21, 22].

All specimens were prepared by removing the skin and muscle attachments while preserving the patella, quadriceps tendon, and patellar tendon for specimen mounting. The preparation process was meticulous to ensure that the specimens were in the best possible condition for testing. The cruciate ligaments, collateral ligaments, and menisci were carefully excised to eliminate any confounding factors during testing.

In real-life in-vivo situations, the direction and nature of patellar fractures can vary, often presenting with multiple crack branches and irregular patterns. However, for the purposes of standardization in our study, we replicated a uniform, single-line fracture across all specimens. By creating a consistent transverse cut at the exact midpoint of the patella's longitudinal length, we ensured that each specimen had a standardized and uniform fracture.

The selection of the patella for each fixation method was carried out using a randomized allocation process. We employed a computerized random number generator for unbiased allocation of patellae to different fixation methods. Each patella was assigned a unique identifier, ensuring equal probability of being allocated to either method. The surgical procedures for both fixation methods were performed by experienced orthopedic surgeons following established protocols. Half of the specimens were repaired using the cannulated screw with VA LNP, while the other half was fixed using TBW.

#### Fixation method

**Fig. 1:**
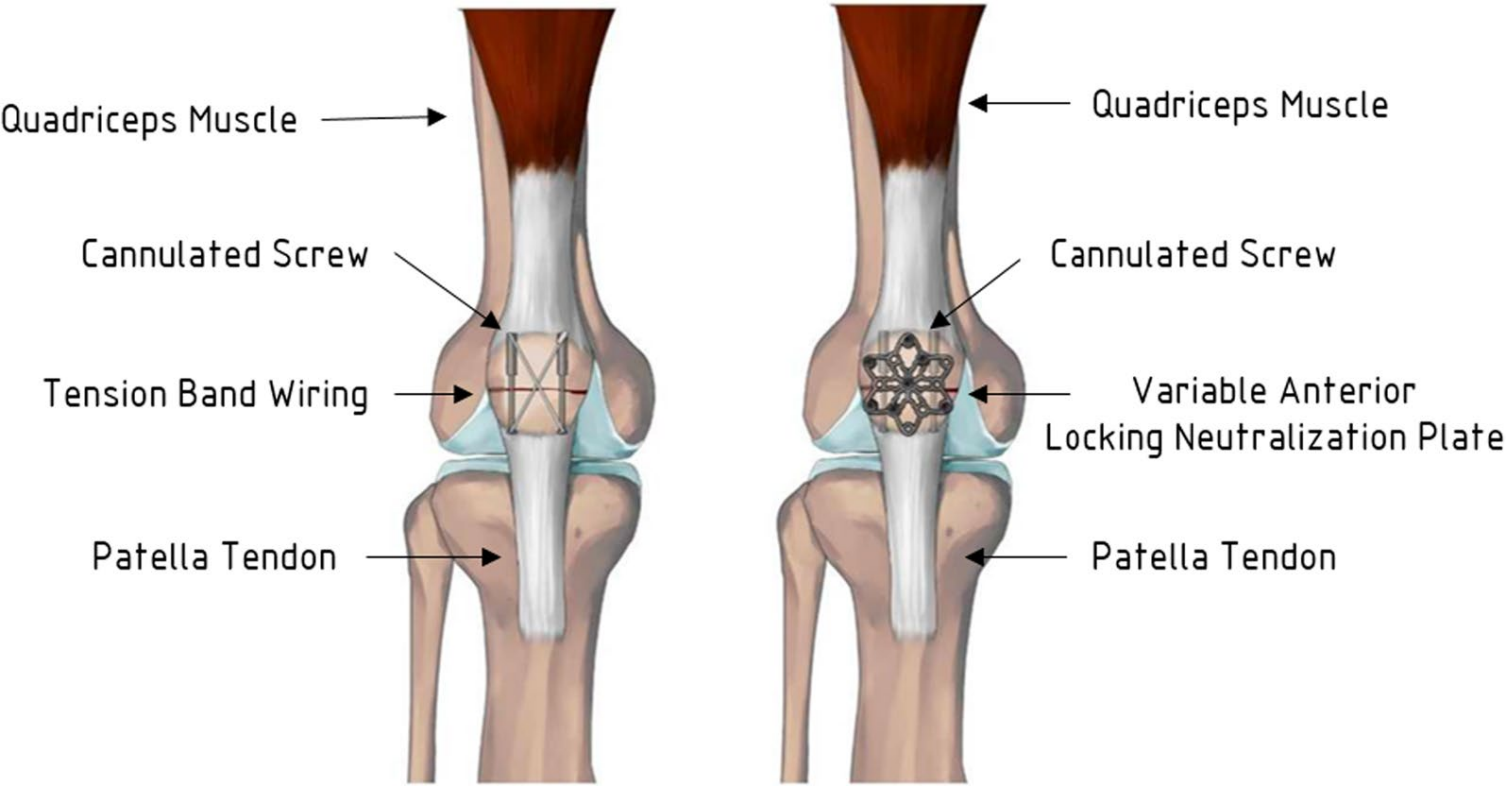
Patella and Quadriceps Tendon Implanted with Cannulated Screw with (left) VA LNP versus (right) TBW

Two distinct fixation techniques were employed to address patella fractures in this study. The first method, TBW with cannulated screws, is a conventional and routinely used technique, offering stability for most patients with bone fractures. It involves minimum tissue disruptions with precise alignment, achieved using large, pointed reduction forceps designed to provide a firm grip and alignment, with serrated jaws for enhanced stability. Preliminary reduction was achieved with 1.25 mm diameter K-wires. Subsequently, stainless steel partially threaded 3.5 mm cannulated screws (DePuy Synthes, Switzerland), ranging from 24 to 34 mm in length, were inserted. The tension band wiring technique involved retrograde inserting a 16G stainless steel wire through the 3.5 mm cannulated screws, traversing the anterior patellar aspect. Soft tissue clearance from the wires was performed precisely, and symmetrical tightening was achieved through twisting with pliers. The second method, employing VA LNP with cannulated screw, is particularly advantageous for osteoporotic or older patients due to its sturdier design, which contributes to greater stability in the construct. This technique used stainless steel 2.7 mm VA LNP plates, contoured to achieve a seamless fit against the anterior patellar cortex. These plates were secured using seven 2.7 mm locking screws distributed in proximal and distal segments (Fig. 1).

#### Cyclic testing

To assess the durability of the VA LNP and TBW fixation for patellar fractures, a cyclic test was conducted using a customized rig and LabVIEW software (National Instruments™, Austin, Texas, USA). The rig was designed to replicate knee movement during full extension, followed by 90° of flexion. To simulate patellar movement, each specimen was secured to the rig with several knots using strings attached to the quadriceps and the end of the patellar ligament. To prevent slippage, the knots were tied multiple times, positioned about 3 inches toward the patella from both ends of the patella tendon. These strings were then connected to a servo-hydraulic shaft, which ensured the patella could be dragged from right to left, creating the appropriate angle.

**Fig. 2:**
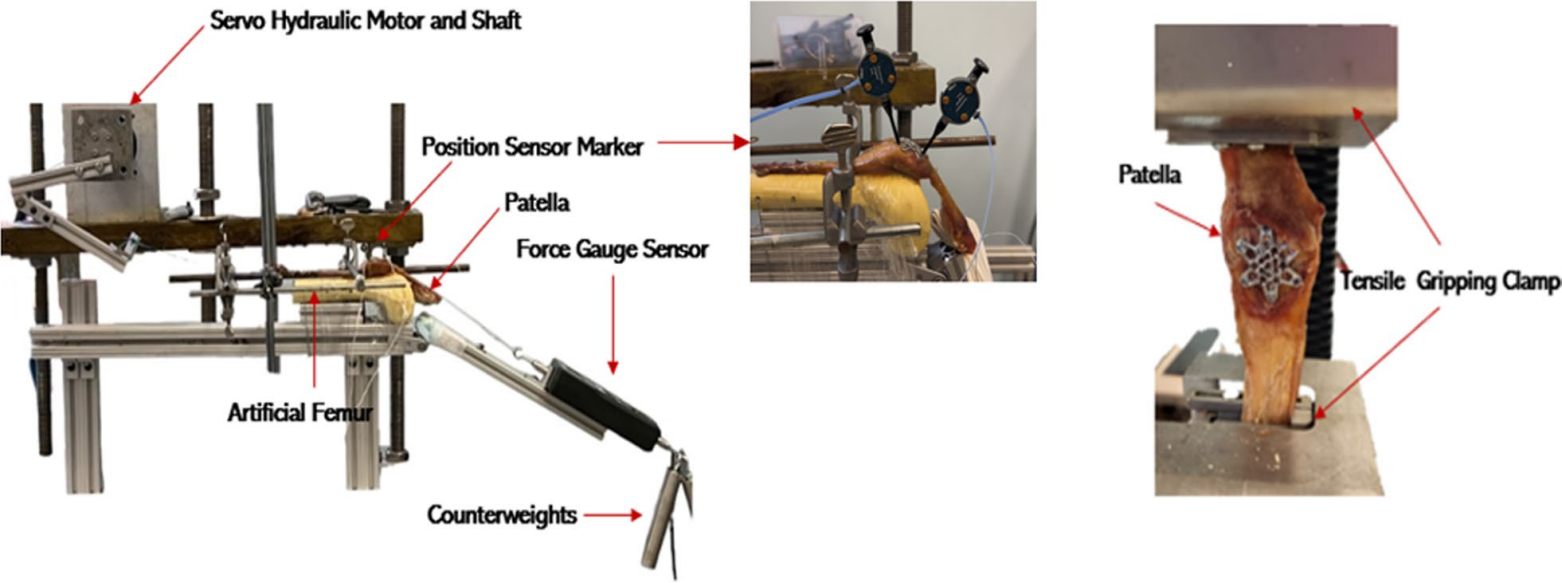
Experimental Setups for (left) Cyclic Testing Rig Configuration, and (right) Tensile Testing Setup Arrangement

To apply a cyclic load to the specimens, aluminum wires were attached to the end of the patellar ligament, and these wires held two bars weighing a total of 1 kg to replicate the appropriate load of the lower leg and foot during standing. The total weight, including the aluminum support, is 3.1 kg. This weight corresponds to the weight of the foot and leg and provides an accurate simulation of the mechanical load on the patella. This method used aligns with the approach detailed in an earlier publication [23]. In that publication, a 3.1 kg weight was placed 25 cm away from the tibiofemoral joint to create a moment at the knee similar in magnitude to that experienced naturally. To ensure the physiological relevance of the test, the specimen was subjected to a load for 500 cycles. Each cycle consisted of an 8-s loading phase followed by an 8-s unloading phase. This loading pattern was designed to replicate the typical loading experienced by the knee joint during daily activities [24]. The displacement and force were recorded during testing using a digital data acquisition system (Fig. 2). Additionally, the motor speed was set at 1/16 cycles per second, a parameter chosen to emulate the slow, controlled movement of the knee during activities such as walking [25].

NDI Optotrak (NDI, Waterloo, ON, Canada) 7mm diameter IRED (Infralight-emitting Diode) encased markers were utilized to precisely measure the gap between the patella fragments at 0.01s intervals throughout the testing period, ensuring continuous monitoring and data recording during the entire experiment. These markers were placed on the patella, one above and one below the partial cut, and attached to the NDI system control unit via the marker strobe. The position sensor was positioned facing the markers, allowing the detection of the infrared light emission and an accurate and precise measurement of their displacement at various points during the cyclic loading.

#### Tensile testing

Patellar failure testing was performed using an electromechanical material testing frame (MTS Systems, Eden Prairie, MN, USA) with a crosshead speed of 1mm/sec and a data acquisition rate of 10 Hz. Tensile gripping clamps were used on the specimen’s top and bottom. The specimens were carefully placed in the clamps, with the quadriceps tendon and patellar ligament secured tightly into the machine to prevent slipping. The grip was clamped around 2/3 of the quadriceps tendon and patella ligament to ensure the load was evenly distributed during testing. The testing machine had a sensor that automatically detected the load and stopped the testing when the failure occurred. We determined failure when the fracture gap becomes 2mm or more [23]. A gap of this size is indicative of a loss of bone stability, which can lead to non-union or delayed healing in fractures.

During testing, a data frequency of 10 Hz was used to capture the load–displacement response of the specimen. The load was applied gradually until the specimen failed. The displacement was recorded using a digital displacement sensor, and the load was recorded using a load cell. Before the tensile testing, each tendon's width, height, and thickness were measured to determine their dimensions. The testing was conducted in a room temperature environment, with regular moistening of specimens using a normal saline spray to prevent drying. To ensure the accuracy and reliability of the results, all specimens were tested under the same conditions, including the same load rate and grip positions.

#### Data analysis

500 flexion—extension cycles were executed for each patella under investigation in the experimental procedure. During the extension cycles, the positions of markers placed on the patella were meticulously recorded at regular intervals of 0.01 s.

The distance between the two markers was calculated based on their recorded 3D coordinates at each point to quantify the gap opening during cycling. To streamline the subsequent data analysis, batches of 100 cycles were combined and evaluated. Within each set of 100 cycles, the maximum and minimum values of the distance were identified, and a single gap measurement was computed using these extremal values. Consequently, 5 gap values were recorded for each patella, enabling the observation of the gap's evolution throughout the cycling process. Each patella sample was subjected to tensile testing using the MTS, and the failure force was determined from the recorded force–displacement curve.

#### Statistical analysis

Given the inherent anatomical variability among individual cadaveric specimens, our study employed a statistical approach for the experimental data. The Shapiro–Wilk test assessed the normality of data distribution within each group. To further ensure the integrity of our data, we incorporated the Grubbs' test to identify any potential outliers that could skew our results. Identified outliers were then excluded to maintain the accuracy of our analysis. Subsequently, we applied Kruskai-Walli’s test to determine the significance of observed differences among multiple groups. To identify specific inter-group differences and statistically significant variations, post-hoc analysis was conducted using the Mann–Whitney U test.

### Finite element modeling

Finite element analysis (FEA) simulations were selectively performed solely for the tensile testing of the patella because the tensile loading condition offers a simpler mechanical environment than cyclic loading. The latter involves complex loading patterns and varying magnitudes of loads over time, thus requiring a more intricate modeling process. In this study, tensile testing was chosen as a suitable starting point for FEA simulations due to its feasibility, accuracy, and high reliability, with the potential for further analysis of more complex loading conditions in future studies. This approach allowed us to generate detailed insights into the mechanical behavior of the patella during tensile loading, optimizing fixation techniques for patellar fractures and ultimately contributing to better patient outcomes.

A 3D solid model of the patella was created using SpaceClaim (ANSYS Inc., Canonsburg, PA, USA) from high-resolution computed tomography (CT) scans of a healthy adult patella. The DICOM files were first imported into Mimics software (Materialize, Belgium) for geometry segmentation, and the resulting geometries were then imported into SpaceClaim for smoothing and pre-meshing. The patellar and quadriceps tendons were manually created in the software based on the dimensions measured during the experiment, as these geometries were challenging to obtain from CT scans. The patellar ligament and quadriceps tendon were created using a series of cross-sectional scans to ensure accuracy. It is important to note that this study utilized a single CAD model, which presents certain limitations in capturing anatomical variability among different specimens.

**Fig. 3:**
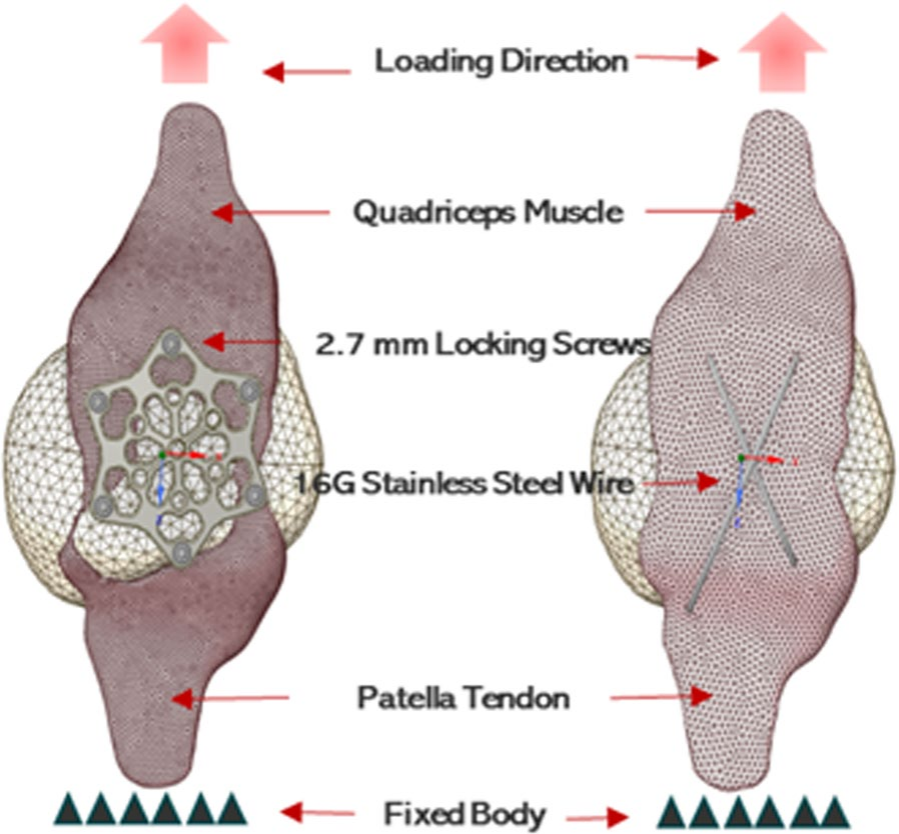
Comparison of 3D CAD Models: VA LNP (left) and TBW (right) for patella and tendon

The model was pre-meshed in SpaceClaim, where a volumetric mesh was generated using a tetrahedral element type. The number of nodes employed in the computational analysis varied between the two fixations, with the VA LNP configuration utilizing 242,526 elements and the wire fixation using 230,941 elements. This divergence in node count can be attributed to addressing convergence issues, necessitating a specific distribution of nodes for accurate and stable simulation outcomes. To simulate the tensile testing, a displacement boundary condition was applied to the bottom surface of the patella model, corresponding to the clamping point used in the experimental testing. A uniform load increment of 100 N was applied in the vertical direction, and the bottom surface of the model was fixed in all directions (Fig. 3).

The material properties assigned to the patella were detailed in Table 1 categorized as linear, isotropic, and homogeneous. The tensile testing simulation was then performed in ANSYS Mechanical (ANSYS Inc., Canonsburg, PA, USA) using the FEA model, and the stress distribution and maximum stress values were analyzed around the wire, plate, and screws to investigate stress concentrations. The contact surface between bone-bone, bone-cartilage, and bone-ligament and bone-screw were configured as bounded interface to simulate natural adherence. Additionally, the interactions between bone-plate and bone-wire were characterized using frictional contact models. The coefficient of friction was assigned a value of 0.3, reflecting the natural roughness of bone surface.

**Table 1:** Young’s Modulus (E), Poisson’s Ratio (ν), and density are provided for each material, including Patella Bone, Quadriceps Tendon, Patella Tendon, cannulated screw, plate, and wire

Material	Density (kg/cm^3)	Young’s modulus (MPa)	Poisson’s ratio
Bone [25]	2000	15,000	0.3
Tendon [37]	1000	200	0.4
Cannulated screw [38]	7750	110,000	0.31
Neutralization plate [39]	4510	120,000	0.36
Tension band wire [40]	2700	69,000	0.34

## Result

A group of sixteen patellae with induced injuries were examined in this study. Among these, eight patellae were treated with VA LNP as prosthetic implants, while the other eight patellae were equipped with TBW. The development of the fracture gap during cyclic testing was monitored, and the force at which failure occurred was recorded. The results for both fixation techniques are provided in the following.

### Experimental testing

#### Cyclic testing

**Fig. 4:**
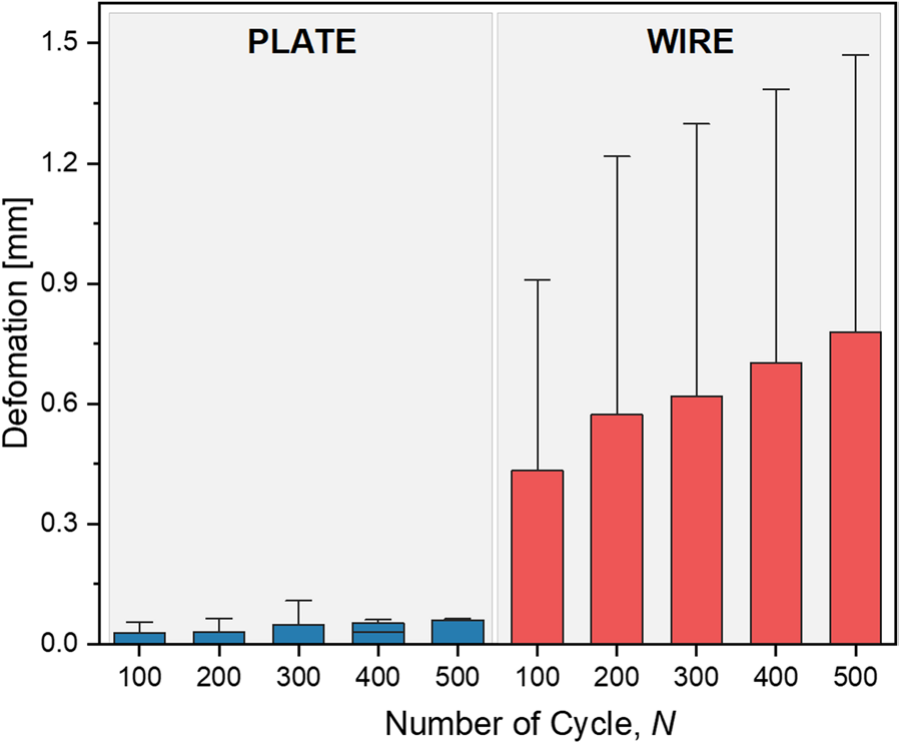
Comparison of average deformation values (in mm) for VA LNP and TBW fixation methods from cycles 100 to 500. The graph demonstrates that within each fixation method group, there is a consistent trend of increasing deformation values as cycles progress, without significant variation between individual cycles

The results of the cyclic testing revealed a noticeable difference in the performance of VA LNP and TBW fixations for patella fractures. The Kruskal–Wallis test was applied to assess deformation values in two different contexts. Firstly, when comparing the overall performance across all tested cycles (100, 200, 300, 400, 500), a significant difference (p = 0.003) was observed between the two fixation groups, the plate group (VA LNP) and the wire group (TBW). This indicates that, overall, these two groups exhibit distinctly different behaviors in response to the cyclic loading during patella fracture testing. However, when we further analyzed the deformation values within each individual group across the different cycles, comparing cycles 100, 200, 300, 400, and 500 within the plate group and within the wire group separately, no significant differences were found (Fig. 4).

It is important to note that in the cyclic testing of patella fractures, one sample from the VA LNP fixation group displayed a distinct pattern of outliers (p = 0.017) compared to the other samples. All data points for this sample were consistently outside the interquartile range, indicating that the deformation values for this particular sample deviated significantly from the rest of the dataset. The data demonstrates the difference in deformation between the two fixation methods, with VA LNP fixation exhibiting approximately 8 times less deformation than TBW fixation at cycle 500.

#### Tensile testing

**Fig. 5:**
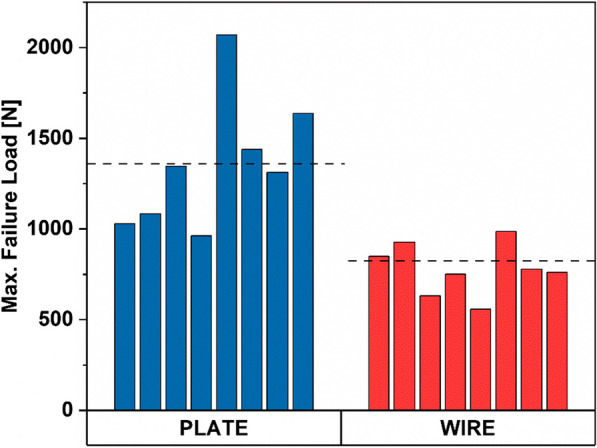
Maximum Failure Force of VA LNP and TBW Fixation Systems during Tensile Testing. The graph shows the maximum failure force for VA LNP and TBW fixation systems during tensile testing. VA LNP fixation demonstrated a higher average maximum failure force than TBW fixation, with VA LNP fixation exhibiting an average maximum failure force of 1359 N, while TBW fixation had a comparatively lower average maximum failure force of 780.1 N

The failure test results further emphasize the differences in the performance of VA LNP and TBW fixations for patella fractures (Fig. 5). The VA LNP fixation samples demonstrated superior performance, with a higher average maximum failure force of 1359 N, while the TBW fixation samples revealed a comparatively lower average maximum failure force of 780.1 N. This suggests that VA LNP fixation provides a significantly higher resistance to failure (p = 0.01) compared to TBW fixation, contributing to the overall stability and durability of the repair. Any delay in fracture fixation may introduce significant alterations in the outcomes, as the bone's intrinsic microhealing processes would have already commenced in the interim.

### Finite element analysis

#### Von Mises stress distribution

**Fig. 6:**
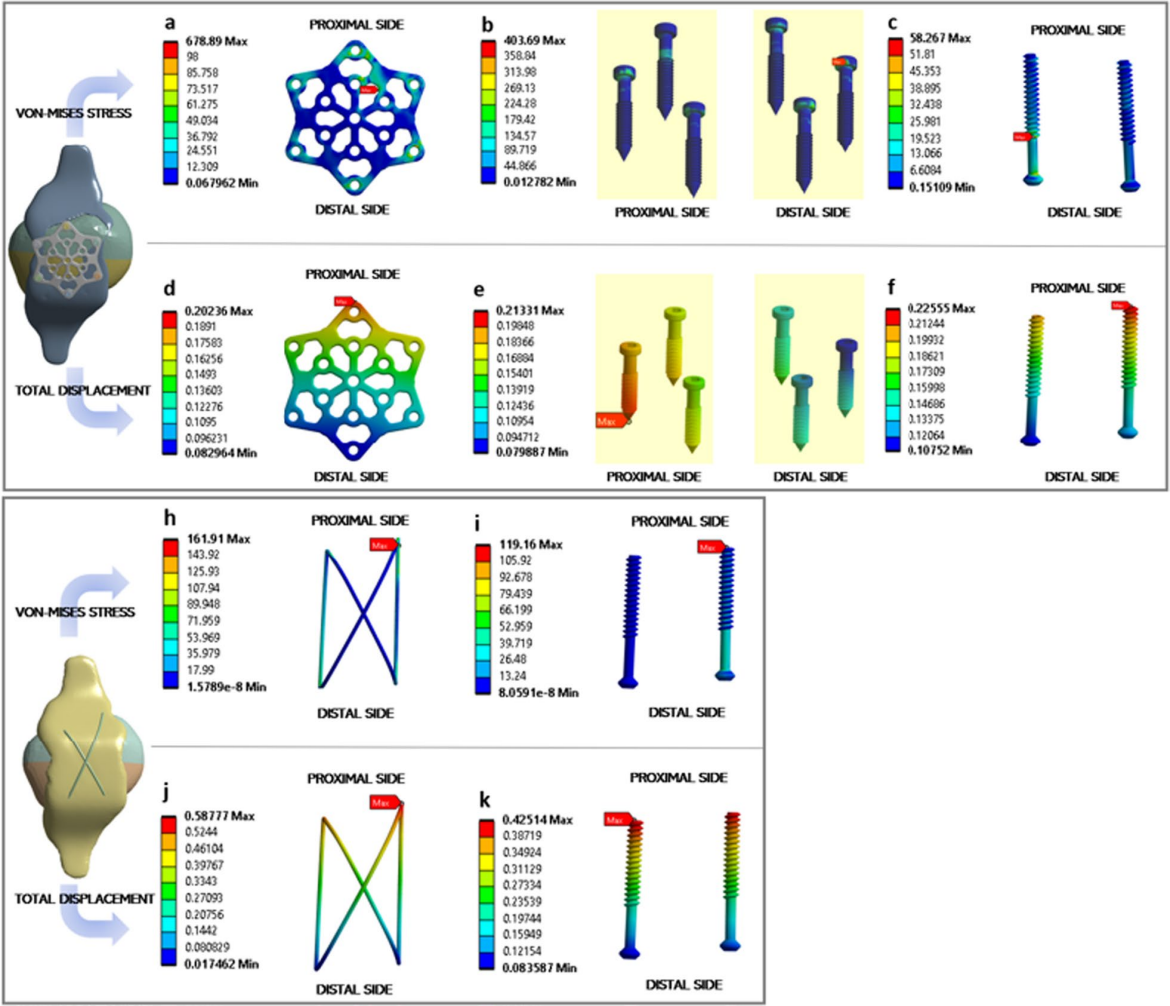
Mechanical analysis results for VA LNP (**a**–**f**) and TBW fixation (**h**–**k**): von-mises stress distribution of VA LNP (**a**–**c**) and TBW (**h**–**i**) along with deformation of VA LNP (**d**–**f**) and TBW (**j**–**k**). Regions highlighted in red indicate maximum stress and displacement, while regions in blue represent minimum force or deformation

The von Mises stress distribution across the VA LNP and TBW fixation (Fig. 6a-f). The analysis highlights distinct stress patterns in various regions of the implant. Notably, a substantial portion of the stress is effectively dissipated by the plate and locking screw, resulting in comparatively lower stress levels exerted on the cannulated screw. This observation holds particular significance, as the primary role of the cannulated screw is to provide structural support to the two fractures rather than actively absorbing stress originating from bone movement. Notably, the peak stress concentration manifests prominently toward the central region of the distal end.

On the other hand, the TBW fixation displays the peak stress at the distal end, with a more pronounced concentration toward the lateral side. The stress concentration, reaching a peak of 678.89 MPa for the VA LNP and 161.91 MPa for the TBW, underscores the notable difference in stress magnitudes. In the case of the TBW fixation method, the observed stress distribution is indicative of its unique mechanical behavior. The peak stress concentration at the distal end is primarily attributable to the tensile forces the tension band wires apply. When adequately tensioned and anchored, these wires convert the applied forces into compressive stresses at the fracture site, promoting stability and healing.

#### Tensile deformation

The VA LNP and TBW fixation response to the applied tensile load is detailed in Fig. 6h–k. In the case of the VA LNP, deformations of the plate, locking screw, and cannulated screws are measured at 0.202 mm, 0.213 mm, and 0.225 mm, respectively. On the other hand, within the TBW fixation method, the wire experiences a deformation of 0.587 mm, while the cannulated screw displays a deformation of 0.425 mm. Notably, the overall deformation of the VA LNP, including the tendon and patella, measured 3.21 mm, accounting for approximately 6.23% of the total deformation. On the other hand, TBW fixation contributed 19.0% of the total deformation, which amounted to 3.05 mm.

#### Stress distribution and deformation around Crack

**Fig. 7:**
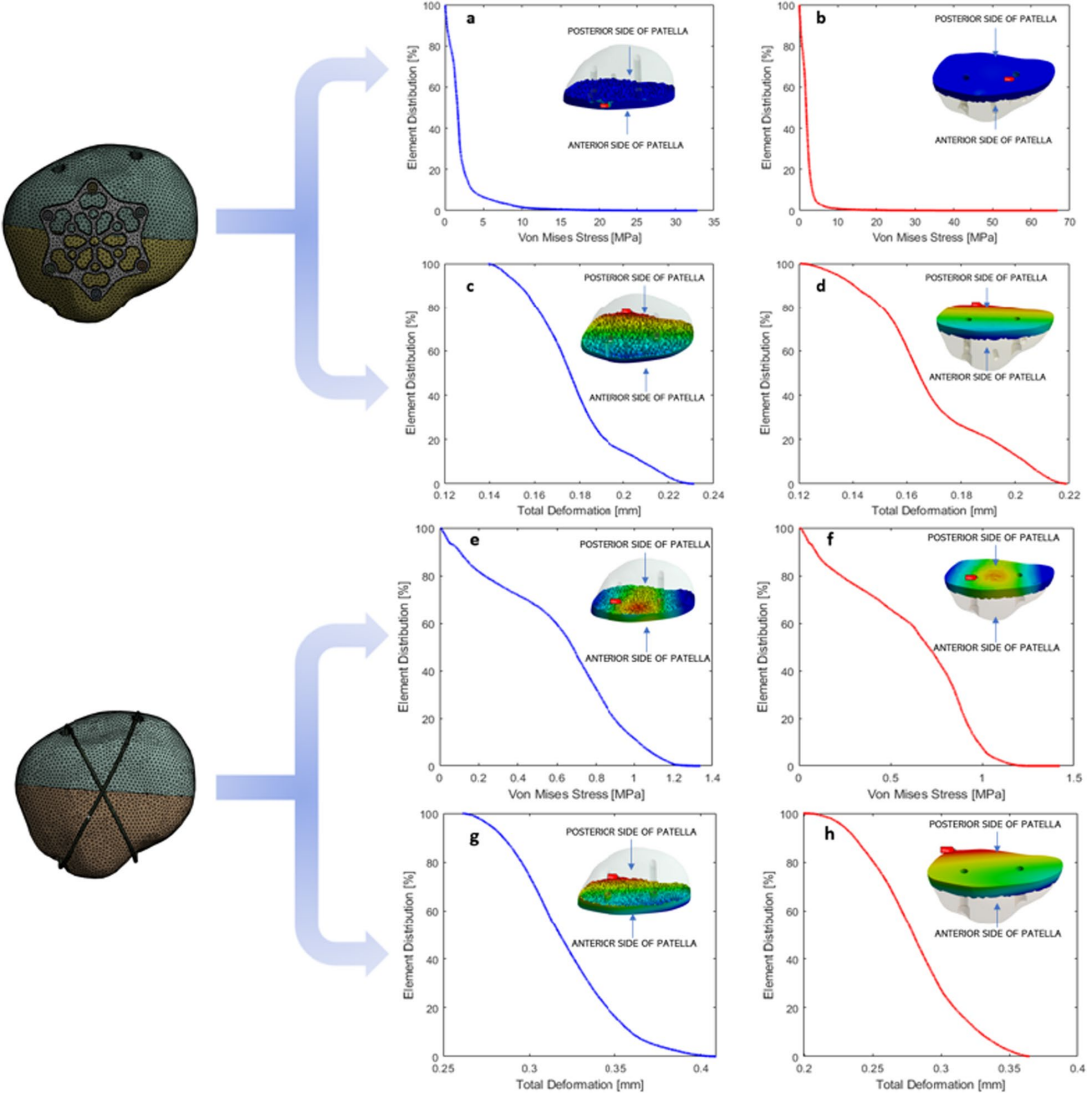
Element distribution of Von-mises stress and total deformation for VA LNP (**a**–**d**) and TBW (**e**–**h**). Red highlights areas of highest stress or deformation, while blue indicates the lowest stress or deformation

In the case of VA LNP fixation, stress primarily concentrates around the cannulated screw hole, peaking at 33.5 MPa on the proximal patella side, with higher stress levels also observed at the distal end around the screw hole (Fig. 7a–d). Deformation in VA LNP is consistent, with the highest value of 0.22 mm at the patella's posterior side. In contrast, TBW fixation exhibits lower stress levels, with 1.38 MPa at the proximal patella side and 1.42 MPa at the distal end (Fig. 7e–h). While TBW shows a reversed stress pattern, it is important to note that the overall stress magnitude is substantially lower than that of VA LNP. Regarding deformation, TBW shows slightly higher values than VA LNP. The location of the highest deformation remains consistent with VA LNP, occurring on the posterior side. However, the overall deformation is slightly more in TBW, measuring 0.43 mm for the proximal side and 0.36 mm for the distal end.

#### Comparison of deformation of FEA with experiment

**Fig. 8:**
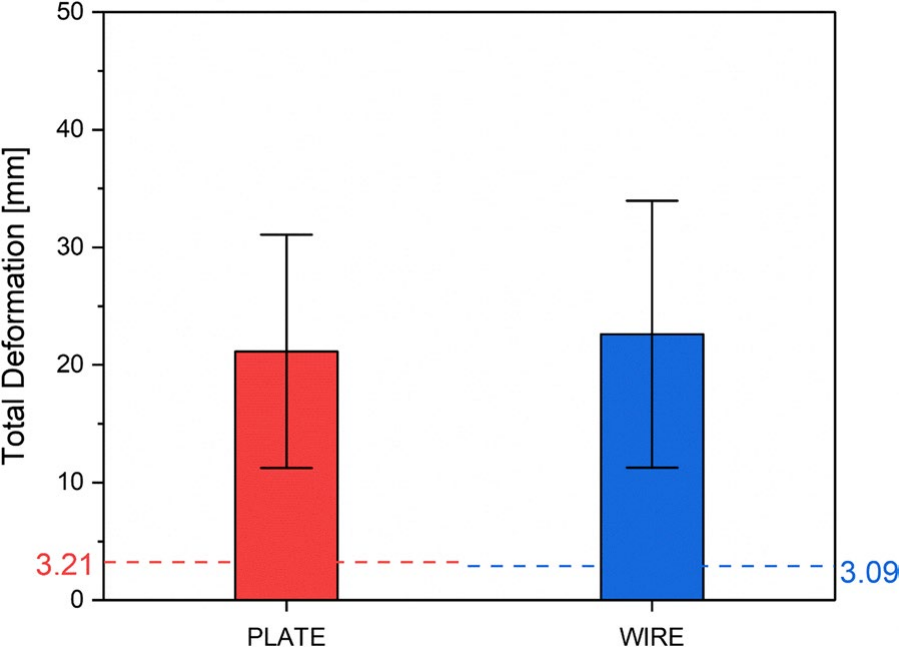
Based on experimental data, the deformation results showcased VA LNP with an average deformation of 21.1 ± 12.2 mm and TBW with 22.6 ± 11.3 mm. The dashed lines represent FEA results, indicating a deformation of 3.21 mm for VA LNP and 3.09 mm for TBW

A marked disparity is evident in the total deformation between the experimental and FEA models. The experimental findings indicated that VA LNP exhibited an average deformation of 22.6 ± 11.3 mm, while the FEA analysis recorded a total deformation of 3.21 mm. Similarly, TBW showed an average deformation of 22.6 ± 11.3 mm, with the FEA model registering a total deformation of 3.09 mm (Fig. 8). This marked contrast can be attributed to the assumptions inherent in the FEA modeling process, which relies on a linear elastic criterion and does not consider plasticity, crack initiation, or propagation. These assumptions were made to maintain consistency in the force control variable, facilitating a direct comparison of deformation between the two materials. This discrepancy can be attributed to the assumption made during the FEA modeling process, which assumes a linear elastic criterion without accounting for plasticity, crack initiation, or propagation.

## Discussion

The challenges associated with fixing patella fractures are often tied to issues with hardware causing discomfort. Moreover, achieving satisfactory functional results remains challenging, even when patients avoid additional surgeries. Using VA LNP could offer a new approach to improving desired outcomes.

This study aims to determine whether VA LNP could be a viable treatment option for transverse patella fractures, surpassing the current gold standard approach involving the TBW technique [26, 27]. DePuy Synthes developed the three holes and six holes VA LNP options to accommodate the various morphology of the patella. VA LNP plate has arms and body. There is also an option of three legs, which gives the surgeon more options to fix the various types of patella fractures.

The advantageous mechanical properties of VA LNPs for patella fractures have been consistently demonstrated [12, 19, 28]. Fixed-angle plates, in particular, have been proven to provide better biomechanical support and reduce the widening of fractures during movement compared to using cannulated screws with the VA LNP technique [29]. Recent designs of VA LNP are believed to offer enhanced adjustability and stability [13, 14, 30]. Stoffel et al. studied the effectiveness of using TBW versus using VA LNP in cadaver tests, and the VA LNP approach showed significantly less displacement after cycling compared to the tension band wiring method [14]. Similar findings were reported by Kfuri et al. [30] However, there is a lack of data on the effectiveness of these VA LNPs for simple transverse patella fractures.

The study demonstrates that the VA LNP and TBW implant achieve displacement rates within the acceptable range for non-operative management in a controlled environment. Nevertheless, after 500 cycles, a significant difference emerges between the two methods. The VA LNP has an average displacement of 0.09 ± 0.13 mm, while the TBW has an average displacement of 0.78 ± 0.13 mm. Despite this divergence, both groups fall within the range of less than 2 mm for proper healing [31], with the VA LNP showing static superiority. However, the marginal clinical significance of this difference might not justify the added expense [31].

The observation that there was no significant difference in deformation within each implant group (VA LNP and TBW) across the cycles ranging from 100 to 500 suggests that both implants demonstrate stable mechanical behavior under the tested conditions, indicating their resilience and reliability under up to 500 cycles of load. This stability, however, also implies that the testing parameters, specifically the number of cycles, may not be extensive enough to fully delineate the mechanical endurance or fatigue limits of these implants. It raises the possibility that conducting tests over a larger number of cycles could reveal more pronounced differences and provide deeper insights into each implant's long-term performance and durability.

Previous studies revealed that the most force the patella tendon could withstand was around 300 N [32]. This value is notably lower than the minimum measurement recorded for either group in our study and significantly below the average load to failure observed for the VA LNP (1359 N) and the TBW (780 N). Notably, a tensile force 780N, achieved through TBW and cannulated screw fixation, is clinically sufficient. However, the VA LNP plate exhibits an almost doubled tensile force capacity at approximately 1359N. We recommend reserving the VA LNP plate for patients with higher body mass or musculature or in instances where the quality of the implant material is debatable and there is a desire for enhanced fixation stability.

Both fixation methods provide increased stability, facilitating the commencement of active quadriceps range of motion at an earlier stage after surgery [33]. However, balancing implementing aggressive postoperative protocols and the potential risk of compromising fixation integrity is crucial. The fixation technique investigated in this study might alleviate this concern, thereby rendering the adoption of more assertive postoperative approaches more viable.

The implant profile becomes a significant consideration when assessing implants, particularly given the prevalence of symptomatic hardware. A meta-analysis by Dy et al. revealed a reoperation rate of 33.6%, primarily due to symptomatic hardware [34]. Lower profile implants, such as the VA LNP, could potentially reduce the incidence of symptomatic hardware and associated complications, even though the higher cost might not justify the marginal decrease in implant removal rates.

Furthermore, delving into our study's Finite Element Analysis (FEA) aspect is essential, as it adds a significant layer of understanding. FEA has emerged as a crucial tool in biomechanical studies, enabling us to gain profound insights into the intricate mechanical behaviors of complex structures. Within this context, it is important to recognize the distinctive mechanical implications associated with the VA LNP configuration. This method typically involves direct attachment to the bone, exerting a unique mechanical influence on the surrounding tissue. In contrast, TBW fixation often rests atop the tendon. This disparity in mechanical behavior led to minor tearing in the tendon with the VA LNP model, a phenomenon that aligns with clinical observations and underscores the importance of accurately representing the mechanics of each fixation method in our simulations.

The stress concentration points vary: the VA LNP exhibits higher stress levels concentrated toward its middle distal end, while the TBW fixation concentrates stress on its lateral side. These variations suggest diverse load-bearing capacities and potential complication zones. Deformation patterns also differ, with VA LNP showing distal-end deformation and the TBW fixation revealing proximal-end deformation. However, it is essential to acknowledge that the FEA tensile test may not fully reflect the loosening and deformation changes experienced during cyclic tests, which could account for the observed discrepancies in deformation between the two methods. These discrepancies may also stem from various factors, including differences in different anatomy models, material properties, and the simplifications inherent in our computational model.

Acknowledging these discrepancies in our FEA model, particularly concerning the deformation patterns and stress concentrations between the VA LNP and TBW methods, we were compelled to initiate a validation process. This process was pivotal in ensuring that our model's outputs closely mirrored real-world tendon behaviors. To this end, we performed a comparative analysis, aligning our model's outputs with experimental biomechanical data. A key part of this analysis involved conducting a sensitivity analysis, wherein we carefully adjusted and examined the impact of various parameters, including material properties and boundary conditions. Furthermore, we aligned our model's predictions with clinical observations and existing biomechanical literature [17, 35, 36].

The study's limitations are associated with its reliance on in vitro examination of fixation techniques, which excludes clinical aspects such as biological healing response, soft tissue contributions, and individual patient factors. Furthermore, the exclusion of a sample, specifically one that exhibited a distinct pattern of displacement during cyclic testing and experienced catastrophic failure below 400 N, raises concerns about tissue quality owing to the absence of bone mineral density measurements. Additionally, using isolated patellae in our study, tested by placing each patella over standard sawbones and a customized rig, is another limitation. Without the context of a knee joint, the patellae may not have entirely replicated the exact physiologic conditions, further affecting the generalizability of our findings. Moreover, incorporating diverse methodologies from existing literature examining patella fracture fixation methods hinders direct comparisons.

Introducing a range of material properties into FEA can significantly refine and potentially alter the result. When analyzing the VA LNP and TBW fixation, these failure criteria can unveil more intricate and realistic stress distribution and deformation patterns. The inclusion of plasticity, for example, can indicate areas of material yielding under high stress not just limited to the middle distal end of the VA LNP and lateral side of the TBW fixation. Moreover, crack initiation and propagation can reveal potential zones of structural weakness that were not previously evident. Additionally, using a diverse range of material properties in the model can lead to a more accurate representation of each structure’s biomechanical behavior and offer new perspectives on their load-bearing capacities and failure mechanisms.

Using a diverse range of material properties in the model can lead to a more accurate representation of each structure’s biomechanical behavior. This offers new perspectives on their load-bearing capacities and failure mechanisms. However, our current approach involves using a homogeneous model to represent bone mechanical properties, which simplifies the modeling process. This approach may not adequately capture the distinct mechanical characteristics of different bone types, such as cortical versus cancellous bone. The homogeneous model's potential limitation lies in its oversimplifying these nuanced differences, which are crucial for a precise depiction of biomechanical behavior.

It is also important to highlight that our FEA model used a customized tendon and ligament based on average values from measured cadaveric specimens, rather than individualized models for each specimen. This approach, while necessary due to limitations in segmenting these structures using available imaging techniques and to ensure a manageable scope for the study, may not fully capture the intricate biomechanical behavior of each specific specimen. Additionally, the limited number of samples used for creating the FEA model is a constraint that could affect the robustness and variability of our findings.

In light of these study results, it is essential to conduct extensive multicenter randomized controlled trials that compare VA LNP fixation with the widely established treatment for patella fractures. Evaluating real patient outcomes is crucial to determine whether the innovative VA LNP reduces rates of symptomatic implants or improves functional outcomes. Additionally, these trials can aid in assessing the cost-effectiveness of adopting the new VA LNP. Furthermore, both fixation techniques observed in the study demonstrated the ability to minimize fracture displacement during cyclic loading, suggesting the feasibility of considering more proactive postoperative management. Future research directions should focus on developing evidence-based guidelines for standardizing postoperative protocols, which have the potential to enhance outcomes by reducing muscle atrophy and reported pain.

## Conclusion

The study evaluated to compare the biomechanical performance of a construct involving cannulated screws with VA LNP with the conventional technique of TBW for treating transverse patella fractures. Experimental testing and FEA were employed to evaluate the implants and gain insights into their mechanical behavior. Outlined below are the primary findings of the study:

1. The VA LNP exhibited significantly lower deformation during cyclic testing than the traditional TBW fixation, highlighting its enhanced stability.
2. In tensile testing, the VA LNP demonstrated a notably higher average maximum failure force than the traditional TBW fixation, indicating improved resistance to failure.
3. Both fixation methods showed displacement within the acceptable range (< 2 mm) for proper healing under cyclic loading conditions.
4. Finite Element Analysis revealed distinct stress patterns for the two fixations, with the VA LNP concentrating stress in the middle of the distal end and the TBW fixation focusing stress at the distal end's lateral side.
5. Deformation patterns also differed, with the VA LNP showing distal-end deformation and the TBW fixation revealing proximal-end deformation.

This study's findings underscore the VA LNP's promising potential in enhancing the treatment of transverse patella fractures. With improved stability, resistance to failure, and biomechanical advantages, this approach is clinically relevant to improving patient outcomes. Future studies could focus on clinical trials to validate the biomechanical findings, further optimizing postoperative protocols and exploring long-term patient benefits.

## Data Availability

Not applicable.
